# C1q/Tumor Necrosis Factor-Related Protein 9: Basics and Therapeutic Potentials

**DOI:** 10.3389/fphys.2022.816218

**Published:** 2022-03-18

**Authors:** Hua Guan, Yanli Wang, Xiangyu Li, Aoqi Xiang, Fengwei Guo, Jianglin Fan, Qi Yu

**Affiliations:** ^1^Shaanxi Key Laboratory of Ischemic Cardiovascular Diseases, Institute of Basic and Translational Medicine, Xi’an Medical University, Xi’an, China; ^2^Department of Pathology, Xi’an Medical University, Xi’an, China; ^3^Department of Cardiovascular Surgery, The First Affiliated Hospital of Xi’an Jiaotong University, Xi’an, China; ^4^Department of Molecular Pathology, Faculty of Medicine, Interdisciplinary Graduate School of Medical Sciences, University of Yamanashi, Chuo, Japan

**Keywords:** CTRP9, cardiovascular disease, atherosclerosis, metabolism disease, type II diabetes mellitus

## Abstract

C1q/tumor necrosis factor-related protein 9 (CTRP9) is a newly discovered adipokine that is the closest paralog of adiponectin. Proteolytic cleavage of CTRP9 leads to the release of the globular domain (gCTRP9), which serves as the major circulating subtype. After binding with adiponectin receptor 1 (AdipoR1) and *N*-cadherin, CTRP9 activates various signaling pathways to regulate glucose and lipid metabolism, vasodilation and cell differentiation. Throughout human development and adult life, CTRP9 controls many biological phenomena. simultaneously, abnormal gene or protein expression of CTRP9 is accompanied by a wide range of human pathological phenomena. In this review, we briefly introduce CTRP9 and its associated signaling pathways and physiological functions, which may be helpful in the understanding of the occurrence of diseases. Moreover, we summarize the broader research prospects of CTRP9 and advances in therapeutic intervention. In recent years, CTRP9 has attracted extensive attention due to its role in the pathogenesis of various diseases, providing further avenues for its exploitation as a potential biomarker or therapeutic target.

## Introduction

**FIGURE 1:**
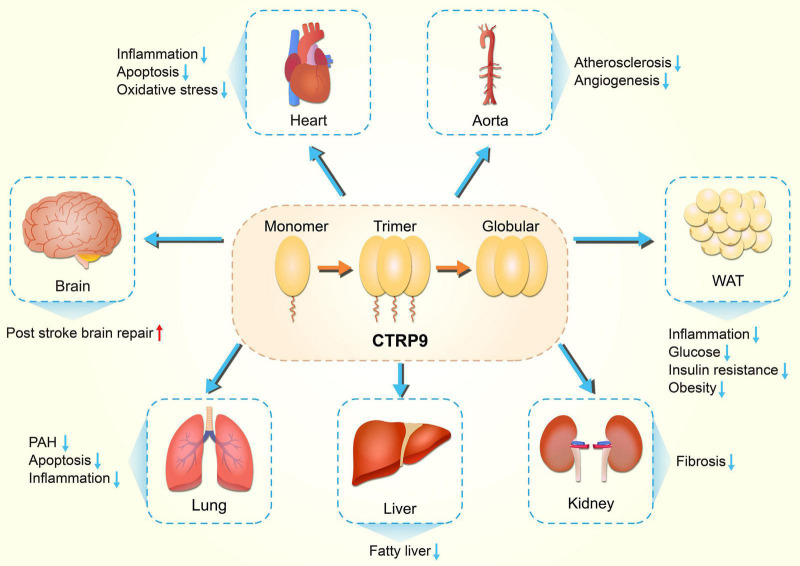
Diversity of physiological functions of CTRP9. CTRP9 protects against various pathologic process, including inflammation, oxidative stress, and apoptosis, in diverse organs. CTRP9, C1q tumor necrosis factor-related protein 9; PAH, Pulmonary arterial hypertension; WAT, white adipose tissue.

Since the discovery of adiponectin in 1995, there has been a fundamental shift in the understanding of adipose tissue (Scherer et al., 1995; Fang and Judd, 2018). Over the past 20 years, many studies have elucidated the physiological functions of adiponectin in obesity, diabetes, inflammation, atherosclerosis and cardiovascular diseases (Wang and Scherer, 2016; Zhao et al., 2021). Adiponectin, which is induced by homologous receptors, inhibits glucose production in the liver, enhances fatty acid oxidation in skeletal muscle, and jointly promotes beneficial metabolism in systemic energy homeostasis (Yanai and Yoshida, 2019). In addition to its role in metabolism, adiponectin also protects cells from apoptosis and reduces inflammation in various cell types through receptor dependent mechanisms (Choi et al., 2020). Adipose tissue is now recognized as an active endocrine organ affecting human health and physiology (Scheja and Heeren, 2019). Wong et al. (2004) discovered the expression of C1q/tumor necrosis factor related proteins (CTRPs) in adipose tissue. To date, 15 CTRPs have been identified (CTRP1-15). They share a common structural domain with adiponectin, which consists of an N-terminal signal peptide, short variable domain, collagen like domain and C-terminal C1q like globular domain (Wong et al., 2004). Both CTRPs and adiponectin belong to the CTRP superfamily. The CTRP superfamily has been shown to have extensive yet opposing effects on lipid metabolism (Seldin et al., 2014), inflammation (Schaffler and Buechler, 2012), apoptosis (Zhang and He, 2019), cardiovascular disease (Shanaki et al., 2020; Si et al., 2020), aging-related disease (Rezabakhsh et al., 2020), and ischemic injury (Yang et al., 2019). In the past decade, CTRP9 has gained increased traction due to its important role in physiological and pathological states (Yang et al., 2016; Figure 1). Here, we present historical views of CTRP9 research over the past decade, discuss the main findings so far, and highlight existing and new issues regarding CTRP9 and its related therapeutic applications.

## The Brief History of C1q Tumor Necrosis Factor Related Proteins

Scherer et al. (1995) first observed the induction of Acrp30/adiponectin (adipocyte complement related protein of 30 kDa) upon adipocyte differentiation, its enhanced secretion by insulin and its structural similarity to the complement factor C1q in 3T3-L1 cells. Maeda et al. (2001) identified a novel mouse cDNA, CORS 26 (collagenous repeat-containing sequence of 26 kDa protein), encoding a secretory protein that later became known as CTRP3 (Wong et al., 2004). Three years later, Wong et al. cloned the adiponectin paralogs from mouse adipose tissue and found that all these proteins contained a C1q-like globular domain, which exhibited striking homology to tumor necrosis factor alpha (TNF-α) based on crystal structure analysis. These proteins were designated as CTRP1-7 (Wong et al., 2004). Peterson et al. (2009) identified CTRP8 and CTRP9 from a human hippocampus cDNAs pool. Evaluation of the exon/intron structure of the *CTRP9* gene on the human chromosome 13q12.12 led to the identification of a nearly identical gene located ∼407 kb upstream of the *CTRP9 gene*. The former was designated as CTRP9A and the latter as CTRP9B (Peterson et al., 2009; Wong et al., 2009). CTRP9A is a secreted protein expressed in mammalian HEK293 cells. In contrast, CTRP9B is expressed intracellularly. When co-expressed with CTRP9A, CTRP9B forms heterotrimers and hetero-oligomeric complexes with CTRP9A, and these complexes are robustly secreted from the cells (Peterson et al., 2009). Subsequently, CTRP10-15 were identified few years later (Bolliger et al., 2011; Wei et al., 2011, 2012, 2013; Seldin et al., 2012).

## Structure Characteristic of CTRP9

**FIGURE 2:**
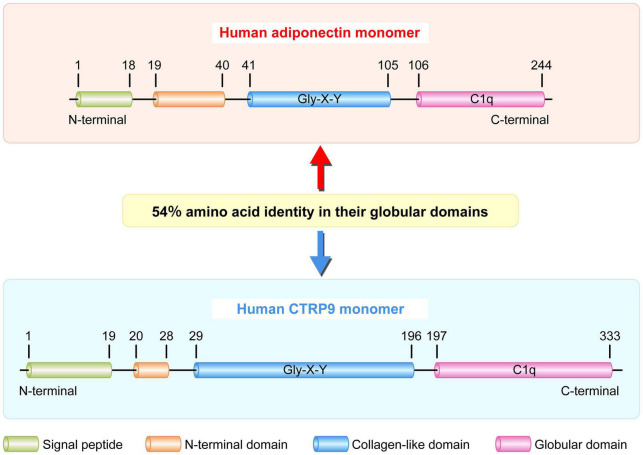
Schematic of human adiponectin and CTRP9 monomer structure. Human adiponectin and CTRP9 monomers are composed of four regions: a signal peptide, an N-terminal domain, a collagen-like domain with multiple Gly-X-Y repeats, and a C-terminal globular domain homologous to the immune complement C1q. These two adipokines share 54% amino acid identity in their globular domains. CTRP9, C1q tumor necrosis factor-related protein 9.

As the closet paralog of adiponectin, CTRP9 is highly conserved throughout evolution. It shares the highest degree of amino acid identify (51%) to the globular domain of adiponectin in mammals and lower vertebrates (Figure 2; Wong et al., 2009; Yang et al., 2017, 2018). Mouse CTRP9 and its corresponding human ortholog share 100, 85, and 89% amino acid identity in their short N-terminal variable regions, collagen-like domain, and C-terminal globular domains, respectively (Chen et al., 2016). CTRP9 is a secreted glycoprotein with multiple post-translational modifications in its collagen domain, which includes hydroxylated prolines and hydroxylated and glycosylated lysines. Additionally, CTRP9/adiponectin complex is another example of a heterotrimer composed of collagen domain-containing proteins with unequal numbers of Gly-X-Y repeats (Wong et al., 2009). CTRP9 monomers form the trimeric complexes, which undergo proteolytic cleavage to release their globular domain (gCTRP9) as the major circulatory isoform (Wong et al., 2009). Moreover, compare to full-length CTRP9, gCTRP9 has stronger effects on the activation of adenosine monophosphate-activated protein kinase (AMPK), AKT serine/threonine kinase (Akt) and endothelial nitric oxide synthase (eNOS) (Zhang et al., 2016). Therefore, promoting gCTRP9 production may be an effective approach for enhancing the biological function of CTRP9. While evaluating the exon/intron structure of the human *CTRP9 gene*, only 7 amino acids were found to be different between CTRP9A and CTRP9B. These amino acid residues Arg-60, Ala-238, Lys-277, Met-281, Gly-290, Val-301, and Pro-333, in CTRP9A were mutated sequentially or in tandem to Cys-60, Val-238, Arg-277, Val-281, Ser-290, Met-301, and Gln-333 in CTRP9B (Peterson et al., 2009).

## The Expression Pattern of CTRP9

CTRP9 is ubiquitously expressed in the human body, highly expressed in fat tissue (relatively higher expressed in stromal vascular fraction), heart, prostate, skin and testis, moderately expressed in the gall bladder, placenta, and urinary ladder, scarcely expressed in the bone marrow, pancreas, and liver (Fagerberg et al., 2014). However, similar to adiponectin, CTRP9 is exclusively expressed in adipose tissue in mice. The expression of *CTRP9* varied with age and sex; CTRP9 shows high expression in female mice as well as significant upregulation in young mice (Wong et al., 2009), suggesting a gender and age-biased expression pattern. The expression of CTRP9 was significantly increased in adipose tissue isolated from 8-weeks-old ob/ob mice compared to that isolated from age-matched wild-type mice, which was distinct from the 12-weeks-old mice (Wong et al., 2009), indicating that the elevated levels of CTRP9 in these young mice may represent a compensatory response before the occurrence of metabolic syndrome. Furthermore, human CTRP9A, but not CTRP9B, is expressed in adipose tissue. CTRP9B is generally expressed at very low levels. In the GenBank database, only two expressed sequence tags correspond to human *CTRP9B* gene; one is derived from the thalamus, while the other is derived from pooled lung and spleen tissue. Of the vertebrate species whose genomes have been sequenced, only humans and primates (chimpanzee and rhesus macaque) carry the *CTRP9B* gene, it is absent in the syntenic region of the mouse genome (Peterson et al., 2009). However, it is unclear whether the polymorphism of *CTRP9* gene will also affect its expression.

## Role of CTRP9 in Metabolic Diseases

### Obesity

To date, various transgenic mouse models have been developed to explore the function of CTRP9. Overexpression of CTRP9 by adenovirus significantly reduced blood glucose and insulin levels in ob/ob mice compared to those in the control group (Wong et al., 2009). To further explore the function of CTRP9, Peterson et al. established CTRP9-overexpressing transgenic mice carrying a HA label. They found that the serum level of CTRP9 in mice was approximately five times higher than that in littermates. This led to a significant increase in fat oxidation in mouse skeletal muscle mitochondria and a decrease in the food intake, body weight, fasting insulin and glucose levels (Peterson et al., 2013). These studies have confirmed that overexpression of CTRP9 can activate the phosphorylation level of AMPK, Akt and mitogen-activated protein kinase (MAPK) in myotubes to regulate insulin resistance (IR) (Zuo et al., 2020b). In contrast, CTRP9 knockout mice were established to explore the physiological and metabolic functions of CTRP9 (Wei et al., 2014). Compared with transgenic overexpression mice, CTRP9-deficient mice displayed significantly increased body weight, food intake and hypothalamic appetite-stimulating neuropeptide levels. Moreover, CTRP9-deficient mice exhibited decreased skeletal muscle AMPK phosphorylation and mitochondrial content, resulting in hepatic steatosis and impaired IR. This observation indicated that mice lacking CTRP9 showed opposing pathological and physiological characteristics (Wei et al., 2014; Zuo et al., 2020b). Furthermore, thromboxane was elevated in genetic and dietary mice models of obesity and diabetes, while thromboxane depletion enhanced insulin sensitivity, glucose homeostasis, and CTRP9 secretion (Lei et al., 2015; Han et al., 2018). All of these data suggest that CTRP9 play a protective role for adipogenesis and contributes to the maintenance of favorable lipid metabolism.

Results of clinical analyses revealed that serum CTRP9 concentration was positively correlated with a favorable glucose metabolic phenotype and the loss of metabolic syndrome, while it was negatively correlated with age, blood pressure, fasting blood glucose levels, IR homeostasis model evaluation, total cholesterol, triglyceride, and low density lipoprotein cholesterol (LDL-C) (Hwang et al., 2014). In contrast, serum CTRP9 levels were significantly higher in patients with obesity than in the lean group, and was down regulated after weight loss surgery, which was inconsistent with previous studies (Wong et al., 2009; Wei et al., 2014), suggesting that CTRP9 may play a compensatory role in obesity (Wolf et al., 2016). Therefore, compared with that in the control group, the expression of CTRP9 mRNA in visceral adipose tissue of patients with obesity was significantly increased and was positively correlated with the steady-state model evaluation of insulin resistance (HOMA-IR) and waist circumference (Masoodian et al., 2020). Obesity, which presents a low-grade inflammatory environment, leads to preeclampsia and adversely affects maternal endothelial function. Therefore, the level of CTRP9 in the obese preeclampsia group was lower than that in the non-obese preeclampsia group, pregnant women with obesity, and pregnant women with a normal body mass index (BMI) pregnant women (Aksin and Andan, 2020). In hence, obesity leads to a decrease in CTRP9 levels and induces the pathogenesis of preeclampsia, with adverse effects on the secretion of IR and plasma glucose.

### Diabetes

CTRP9 have been reported to be associated with metabolic diseases such as obesity and type 2 diabetes mellitus (T2DM). For instance, a clinical study by Bai et al. (2017) indicated that serum CTRP9 levels in patients with T2DM were lower; however, this result was not statistically significantly. Besides, serum CTRP9 levels were significantly correlated with systolic pressure and levels of C-reactive protein and leptin. Moreover, leptin was predictive of serum CTRP9 levels (Bai et al., 2017), which was consistent with the results reported by Fujita et al. (2017) and Spurna et al. (2018). Furthermore, circulating CTRP9 levels are positively correlated with markers of obesity and IR, including BMI, fasting blood glucose level, insulin and LDL-C (Jia et al., 2017). Diabetic retinopathy is a common and specific microvascular complication of diabetes mellitus, along with diabetic neuropathy (Lechner et al., 2017). CTRP9 improved cell viability and attenuated oxidative stress and apoptosis by activating AMPK/nuclear factor, erythroid 2 like 2 pathway in high glucose induced ARPE-19 cells (Cheng et al., 2020), suggesting CTRP9 may be a promising functional target for the treatment and prevention of DR. Otherwise, serum CTRP9 levels decreased significantly in T2DM patients with pulmonary infection (Zha et al., 2020). Serum levels of CTRP9 during the first trimester of pregnancy were significantly decreased in patients with gestational diabetes mellitus compared with those in healthy individuals and were negatively correlated with the BMI or oral glucose tolerance test (Na and Ji, 2020), which was corresponded to the research performed by Xia et al. (2020). Moreover, diabetic nephropathy, a leading cause of end-stage renal disease, is characterized by excessive accumulation of extracellular matrix, leading to early thickening of the glomerular and tubular basement membrane (Umanath and Lewis, 2018). Hu et al. (2020) revealed that CTRP9 ameliorated renal dysfunction and injury at the structural and functional levels in diabetic db/db mice by inhibiting glomerular and tubular glycogen accumulation, ameliorating hyperglycemia-mediated oxidative stress and apoptosis. High intensity interval training (HIIT) is an effective training method to improve energy metabolism and insulin sensitivity. The concentration of CTRP9 in the body increases significantly after a single HIIT session, suggesting that a single HIIT session may stimulate the secretion of CTRP9 in healthy men (Kon et al., 2019). In diabetes mellitus, the plasma concentration of CTRP9 is negatively correlated with the extent of platelet aggregation. Increased CTRP9 production or exogenous CTRP9 supplementation protects patients with diabetes from cardiovascular damage by reducing aberrant platelet activity (Wang et al., 2016). Collectively, these observations suggest that CTRP9 acts as a biomarker for diabetes and may have great potential to improve glucose metabolism dysfunction.

### Fatty Liver

CTRP9 enhances the phosphorylation of AMPK and mediates the expression of sterol regulatory element binding transcription factor 1 in HpG2 cells. Moreover, CTRP9 exert protective effects on autophagy and endoplasmic reticulum stress (ERS) induced apoptosis to alleviate hepatic steatosis (Jung et al., 2015). However, clinical studies established that there is no significant difference between the serum levels of CTRP9 protein and the development of non-alcoholic fatty liver disease. Furthermore, serum levels of CTRP9 protein are positively correlated with BMI, waist circumference, fasting insulin levels, decreased hemoglobin levels, and HOMA-IR, suggesting that the correlation between CTRP9 and non-alcoholic fatty liver is most likely due to the effect of CTRP9 on obesity (Zhang C. et al., 2018). Thus, promoting CTRP9 production maybe an effective approach for alleviates hepatic steatosis.

### Polycystic Ovary Syndrome

Serum CTRP9 levels of patients with polycystic ovary syndrome (PCOS) exhibited a positive correlation with unfavorable levels of lipids, such as LDL-C and total cholesterol were higher in patients with PCOS compared to these healthy subjects. However, whether the participants suffer from PCOS is not clear. Therefore, abnormal blood lipid levels are the major contributor to abnormal CTRP9 expression (Forouhi et al., 2016), further studies are required to investigate the mechanistically action of CTRP9 in PCOS.

Metabolic disorders include metabolic syndrome, IR, T2D and hyperlipidemia, which may be related to a variety of metabolic pathways (Yoon et al., 2021). Although these diseases have their own physiological symptoms, in this review, we summarize the role and function of CTRP9 in these diseases (Tables 1, 2). These characteristics indicate that CTRP9 is a potential target for treatment of obesity, hepatic steatosis and diabetes. However, more evidence is needed to prove the association between CTRP9 and lipid related diseases. There is no doubt that the next step in clarifying the role of CTRP9 in metabolic syndrome is to apply what we have learned from mouse models to human populations. As studies to determine the conditions for the reduction of CTRP9 concentration are ongoing, future clinical studies may focus on identifying the link between CTRP9 concentration or mutation and lipid related diseases and confirming whether the clinical application of therapeutic drugs for CTRP9 has other unsatisfactory side effects. In addition, the discovery of CTRP9 function is helpful to explore it as one of the biomarkers of metabolic disorders, which may greatly improve the diagnostic accuracy of complex phenotypes.

**TABLE 1:** The clinic studies about the concentration of CTRP9 in patients.

Type of disease	Concentration of CTRP9	*P-*value	Function	Ref.
Aortic calcification in renal allograft recipients	2008: 2.05 [2.00–2.13] ng/mL (*n* = 50); 2016: 2.31 [2.14–2.60] ng/mL (*n* = 50);	*P* < 0.05	CTRP9 is a vascular protective factor in renal allograft recipients.	Miyatake et al., 2020
CAD	Non-CAD: 96.14 ± 33.13 pg/mL (*n* = 121); CAD: 83.89 ± 36.18 pg/mL (*n* = 241);	*P* < 0.05	CTRP9 is an independent protective factor of CAD.	Wang et al., 2015
CAD in Egyptian postmenopausal females	Control: 304.46 ± 9.60 ng/mL (*n* = 13); CAD: 194.90 ± 3.34 ng/mL (*n* = 29); T2DM: 126.76 ± 2.95 ng/mL (*n* = 29); CAD+T2DM: 101.40 ± 5.7 ng/mL (*n* = 15)	*P* < 0.05	CTRP9 showed decreased serum levels in females suffering from CAD, T2D, and CAD secondary to T2D.	Ahmed et al., 2018
CAD with OSA	moderate/severe OSA group: 4.7 [4.1–5.2] ng/mL (*n* = 89); no/mild OSA group: 4.9 [4.4–6.0] ng/mL (*n* = 65)	*P* < 0.05	Plasma CTRP9 levels were independently related to the prevalence of moderate/severe OSA in patients with CAD.	Li et al., 2020
Cerebrovascular stent	With restenosis: 159.64 ± 32.55 ng/mL (*n* = 66); Without restenosis: 184.53 ± 34.53 ng/mL (*n* = 62)	*P* < 0.001	CTRP9 low expression after cerebrovascular stent implantation may increase the risk of in-stent restenosis.	Pan et al., 2020
HFrEF	Healthy subjects; 180.70 ± 51.05 ng/mL (*n* = 176); Heart failure patients: 124.60 ± 37.58 ng/mL (*n* = 168)	*P* < 0.001	CTRP9 are decreased in patients with HFrEF.	Gao et al., 2019
HIV	HIV negative: 0.20 [0.13–2.25] (*n* = 92); HIV positive: 0.17 [0.13–1.87] ng/mL (*n* = 209)	N.S	CTRP9 was not associated with the HIV.	Kasher Meron et al., 2019
MS	Healthy individuals: 0.77 [0.64–1] ng/mL (*n* = 24); MS patients: 0.81 [0.64–0.95] ng/mL (*n* = 26)	N.S	CTRP9 may not be associated with the pathogenesis of MS.	Rasooli Tehrani et al., 2019
NAFLD	Controls: 1.39 ± 0.76 (*n* = 79); NAFLD: 1.79 ± 1.06 (*n* = 82)	N.S	CTRP9 was not the biomarker associated with the pathogenesis of NAFLD.	Zhang C. et al., 2018
Obese and preeclamptic	Normal BMI pregnant: 110 ± 45 ng/mL (*n* = 40); obese pregnant women: 77 ± 24 ng/mL (*n* = 40); non-obese preeclampsia: 107 ± 38 ng/mL (*n* = 40); obese preeclampsia: 42 ± 8 ng/mL (*n* = 40)	*P* < 0.001	Obesity causes a decrease in CTRP9 levels and contributes to the pathogenesis of preeclampsia with adverse effects on the vascular and placental system.	Aksin and Andan, 2020
Obese	Lean group: 76.5 [22.4–120.5] (*n* = 62); Obese group: 115.3 [60.9–254.5] (*n* = 59)	*P* < 0.001	CTRP9 levels are elevated in obesity and significantly decrease following weight loss surgery.	Wolf et al., 2016
PCOS	Control: 5.0 ± 7.6 ng/mL (*n* = 27); PCOS: 8.8 ± 19.9 ng/mL (*n* = 29)	N.S	Similar serum CTRP9 were found in PCOS subjects and controls.	Forouhi et al., 2016
SSc	Control: 19.1 ± 31.0 ng/mL (*n* = 29); SSc: 213.8 ± 685.9 ng/mL (*n* = 126)	*P* < 0.001	Elevated CTRP9 was associated with SSc and radiologic evidence of lung fibrosis.	Korman et al., 2018
T2DM	Healthy control: 198.6 [43.2–1781.2] (*n* = 21); T2DM: 41.2 [40.4–297.1] (*n* = 56)	N.S	An elevated CTRP9 level in obesity is a compensatory response due to CTRP9 effect (glucose lowering and insulin sensitizing).	Spurna et al., 2018
T2DM	NGT: 69.39 ± 30.56 (*n* = 108); IGT: 94.37 ± 45.21 (*n* = 92); nT2DM: 138.35 ± 52.63 (*n* = 106)	*P* < 0.05	Circulating CTRP9 levels are increased in patients with newly diagnosed type 2 diabetes and correlated with insulin resistance.	Jia et al., 2017
T2DM	T2DM: 12.2 [9.0–136.1] pg/dL (*n* = 28)	N.S	CTRP9 may reflect diabetic renal vascular risk in association with atherosclerosis and abnormal glucose metabolism besides of impaired vaso-relaxation in patients with T2DM.	Fujita et al., 2017
T2DM	Control: 135.4 [111.4–154.0] (*n* = 139); T2DM: 149.0 [108.2–194.0] (*n* = 124); OGTT for 0 h: 149.0 [108.2–194.0] (*n* = 124); OGTT for 2 h: 150.5 [64.9–235.5] (*n* = 124)	N.S	Leptin was predictive of serum CTRP9. CTRP9 was not regulated by plasma glucose.	Bai et al., 2017
T2DM	Normal glucose tolerance: 415.0 ± 122.8 ng mL (*n* = 120); Prediabetes or T2DM: 276.3 ± 1270.2 ng/mL (*n* = 101)	*P* < 0.01	Serum CTRP9 concentrations were positively associated with favorable glucose or metabolic phenotypes.	Hwang et al., 2014
T2DM and CAD	Control: 148.7 ± 4.0 ng/mL (*n* = 80); CAD:202.0 ± 4.9 ng/mL (*n* = 157); T2DM:191.4 ± 10.1 ng/mL (*n* = 37); CAD+T2DM:211.2 ± 6.8 ng/mL (*n* = 63)	*P* < 0.01	CTRP9 was a compensatory response to insulin resistance, inflammatory milieu and endothelial dysfunction	Moradi et al., 2018
T2DM with CKD	Non-CKD: 13.9 [8.2–22.6] μg/mL (*n* = 29); CKD: 22.5 [14.9–37.5] μg/mL (*n* = 29)	*P* < 0.001	CTRP9 is associated with atherosclerosis in diabetic patients without CKD.	Asada et al., 2016

*CAD, coronary heart disease; CKD, chronic kidney disease; HFrEF, heart failure with reduced ejection fraction; HIV, human immunodeficiency virus; MS, multiple sclerosis; NAFLD, nonalcoholic fatty liver disease; OSA, obstructive sleep apnea; PCOS, polycystic ovary syndrome; SSc, Systemic sclerosis; T2DM, type II diabetes mellitus; N.S, non-significant.*

**TABLE 2:** The experimental study characteristics of CTRP9 in metabolic disease.

Disease	Type of study	Materials	Function and molecular mechanism	References
Obesity	(1) Animal	(1) CTRP9 transgenic mice; Ob/ob mice; C2C12 cells; 3T3-L1 cells	(1) Activate AMPK, AKT and p44/42 MAPK signaling pathway in cultured myotube; overexpression CTRP9 lower serum glucose levels.	Wong et al., 2009
	(2) Animal/ Cell line	(2) CTRP9 transgenic mice; WT mice; Rat H4IIE hepatocytes and rat L6 myocytes	(2) Protects from diet-induced obesity and metabolic dysfunction, enhanced fat oxidation in L6 myotubes via AMPK activation and reduced lipid accumulation in H4IIE hepatocytes.	Peterson et al., 2013
	(3) Animal	(3) CTRP9 KO mice; WT mice	(3) Control energy balance via central and peripheral mechanism, including reduced skeletal muscle AMPK activation and mitochondrial content.	Wei et al., 2014; Zuo et al., 2020b
	(4) Animal	(4) Male WT mice, ob/ob mice, and Tbxas KO mice	(4) Lose of Tbxas was correlated with the up-regulation of CTRP9.	Lei et al., 2015
	(5) Animal	(5) WT mice feed a high fat diet	(5) Improves the anti-contractile effects of perivascular adipose tissue via the AMPK-eNOS pathway in diet-induced obese mice	Han et al., 2018
Diabetes	(1) Cell line	(1) ARPE-19 cells	(1) Attenuates HG-induced oxidative damage and apoptosis in ARPE-19 cells.	Cheng et al., 2020
	(2) Animal	(2) Db/db mice	(2) Ameliorated renal dysfunction and injury at the structural and functional level in diabetic db/db mice	Hu et al., 2020
Fatty liver	(1) Animal/ Cell line	(1) WT mice feed a high fat diet; HepG2 cells	(1) Alleviates hepatic steatosis through relief of ER stress via the AMPK-mediated induction of autophagy.	Jung et al., 2015

*AKT, AKT Serine/Threonine Kinase; AMPK, AMP-activated protein kinase; eNOS, endothelial nitric oxide synthase; ER, endoplasmic reticulum; HG, high glucose; KO, knock out; MAPK, mitogen-Activated Protein Kinase; WT, wild type.*

## Role of CTRP9 in Cardiovascular Disease

### Angiogenesis

CTRP9 upregulates the expression of AdipoR1 in a concentration dependent manner, subsequently activates AMPK phosphorylation, and enhances eNOS transcription to accelerate nitric oxide production stimulating vasodilation of the aortic rings (Zheng et al., 2011; Yamaguchi et al., 2020). In addition, CTRP9 inhibits the growth of vascular smooth muscle cells (VSMCs) through a protein kinase A dependent mechanism protecting against injury. This inhibition leads to a reduction in the formation of neointima in the model of steel wire arterial injury (Uemura et al., 2013), suggesting that the therapeutic approaches to enhance CTRP9 production could be valuable for prevention of vascular restenosis.

### Myocardial Injury

Adiponectin was shown to improve cardiomyocyte contractile function in db/db diabetic obese mice and high fat diet (HFD)-induced obesity mice, suggesting that adiponectin deficiency might aggravate HFD-induced obesity, metabolic derangement, cardiac hypertrophy, and contractile dysfunction through decreased myocardial autophagy (Dong and Ren, 2009; Guo et al., 2013). CTRP9 is highly similar to adiponectin in terms of structure and function. This section summarizes the physiological effects of CTRP9 on myocardial injury, including myocardial ischemia, myocardial infraction (MI), myocardial fibrosis and hypertrophy. The expression of CTRP9 is decreased in patients with heart failure with reduced ejection fraction. This decrease is correlated with disease severity and increased morbidity and mortality (Gao et al., 2019). Additionally, it was reported that CTRP9 alleviated inflammation, apoptosis by activating AMPK, Nrf2 or protein kinase A-cAMP responsive element binding protein 1 pathway, thereby ameliorating MI or protecting against myocardial ischemia/reperfusion (MI/R) injury in rats (Kambara et al., 2015; Zhao D. et al., 2018; Liu et al., 2020). Therefore, CTRP9 supplementation might be beneficial for the treatment for prevention of various heart diseases including ischemic heart disease.

A series of experimental studies have revealed that CTRP9 plays an important role in the pathogenesis of diabetic heart injury (Tables 1, 3; Kambara et al., 2012; Su et al., 2013; Sun et al., 2013; Yuan et al., 2015; Bai et al., 2016). The incidence of heart failure after MI in patients with T2DM is increasing year by year (Lehrke and Marx, 2017). CTRP9 is highly expressed in myocardium, and its subtype gCTRP9 is formed by post-translational proteolysis modification, which activates AMPK, Akt and eNOS to stimulate a protective mechanism for cardiac survival and reduce diabetic heart injury (Su et al., 2013; Yuan et al., 2015; Bai et al., 2016). In an I/R model of rats fed with HFD, CTRP9 treatment significantly increased the expression of myocardial disulfide bond oxidase-like protein, reduced ERS, and alleviated the diabetes mellitus-induced heart injury (Bai et al., 2016). CTRP9 is also upregulated in adiponectin knockout mice, while it was decreased significantly in db/db mice (Yuan et al., 2015). Consistently, the expression level of CTRP9 was 100-fold higher than that of adiponectin in the heart, while the expression level of CTRP9 was greatly reduced in HFD-induced diabetes mice (Su et al., 2013). Additionally, systemic administration of CTRP9 significantly reduced the infarct size and myocardial apoptosis by activating the AMPK signaling pathway (Kambara et al., 2012), indicating that CTRP9 represents a novel target molecule for manipulation of myocardial ischemic injury.

**TABLE 3:** The experimental study characteristics of CTRP9 in cardiovascular disease.

Disease	Type of study	Materials	Function and molecular mechanism	References
Angiogenesis	(1) Animal	(1) Aortic rings isolated from WT C57BL/6 mice	(1) Exerts vasculoprotective effects via the AdipoR1/AMPK/eNOS dependent/NO mediated signaling pathway.	Zheng et al., 2011
	(2) Animal	(2) WT mice, CTRP9 KO mice, and eNOS-KO mice	(2) Promotes endothelial cell function and ischemia-induced revascularization through the eNOS-dependent mechanism.	Yamaguchi et al., 2020
	(3) Animal	(3) Left femoral arteries of WT mice were injured by a steel wire.	(3) Attenuates neointimal formation following vascular injury through inhibit VSMC growth via cAMP-dependent mechanism.	Uemura et al., 2013
Myocardial injury	(1) Animal	(1) WT, CTRP9 KO mice	(1) Protects against acute cardiac damage by suppressing inflammatory reactions through AdipoR1/AMPK signaling.	Kambara et al., 2015
	(2) Animal	(2) WT, CTRP9 KO mice	(2) Protects against MI/R injury via activation of the PKA-CREB pathway and inhibiting cardiomyocyte apoptosis.	Zhao D. et al., 2018
	(3) Animal	(3) WT rats	(3) Alleviates inflammation to ameliorate myocardial infarction in rats by activating Nrf2.	Liu et al., 2020
	(4) Animal/ cell line	(4) High-fat diet induced type 2 diabetes model mice; H9c2 cardiac muscle cell line	(4) Downregulation of CTRP9 induces TNF-α-initiated oxidative PPARγ suppression contributes to exacerbated diabetic cardiac injury.	Su et al., 2013
	(5) Animal/ cell line	(5) C57BL/6J mice feed a high fat diet; 3T3-L1 cell line	(5) Enhancing cardiac CTRP9 production attenuates diabetic cardiac injury.	Yuan et al., 2015
	(6) Animal/ Primary cell	(6) Mouse model; Primary cardiac myocytes; 3T3-L1 cells	(6) Protects against acute cardiac injury following ischemia- reperfusion via an AMPK-dependent mechanism.	Kambara et al., 2012
	(7) Animal/ cell line	(7) H9c2 cells; Rats feed a high fat diet	(7) Exerts cardioprotection by reducing ERS in diabetic heart through increasing disulfide-bond A oxidoreductase-like protein	Bai et al., 2016
	(8) Animal	(8) Rats with myocardial infarction	(8) Attenuates atrial inflammation and fibrosis via inhibitory effects on the TLR4/NFκB and Smad2/3 signaling pathway.	Liu et al., 2019a
	(9) Animal/ Primary cells	(9) Myocardial infraction rat model; Rat peritoneal macrophages	(9) Modulating M1/M2 macrophage polarization via the TLR/MD2/ MyD88 and AMPK-NFκB pathway.	Liu et al., 2019b
	(10) Animal/ Primary cells	(10) CTRP KO mice; Adult ventricular cardiomyocytes	(10) Promotes hypertrophic cardiac remodeling and dysfunction after TAC in mice and induced hypertrophy in isolated adult cardiomyocytes.	Appari et al., 2017
	(11) Animal/ Primary cells	(11) CTRP9 KO mice; Neonatal rat cardiac myocytes	(11) Anti-myocardial lipotoxicity properties and inhibited cardiac hypertrophy through the LKB1/AMPK signalling pathway.	Zuo et al., 2020b
	(12) Animal/ Cell line	(12) Mice with myocardial infraction; ADSCs	(12) Maintaining a healthy microenvironment facilitating stem cell engraftment in infarcted myocardial tissue.	Yan et al., 2017; Weng et al., 2019; Du et al., 2020
Atherosclerosis	(1) Animal/ Cell line	(1) ApoE KO mice; RAW 264.7 cell	(1) Attenuates the development of atherosclerosis and enhances the plaque stability in ApoE KO mice.	Li et al., 2015; Huang et al., 2019
	(2) Cell line	(2) RAW 264.7 cell	(2) Showed atheroprotective function via CTRP9-AMPK- NLRP3 inflammasome pathway.	Zhang et al., 2019; Chen et al., 2020a
	(3) Cell line	(3) ThP-1 cell	(3) Inhibits THP-1 macrophage foam cell formation by entophagy.	Zhang L. et al., 2018
	(4) Cell line	(4) Endothelial cell	(4) Attenuates palmitic acid-induced endothelial cell senescence via increasing autophagy	Lee et al., 2020
	(5) Cell line	(5) Human aortic VSMCs	(5) Inhibits the cholesterol-induced VSMCs phenotypes switch and cell dysfunction by activating AMP-dependent kinase.	Liu et al., 2017
	(6) Cell co-culture system	(6) ThP-1 cell; VSMCs	(6) Induces macrophages polarization into M1 phenotype through activating JNK pathway and enhances VSMCs apoptosis in macrophages and VSMCs co-culture system.	Chen et al., 2020b
PAH	(1) Animal; Cell line	(1) Human primary pulmonary artery epithelial cells; Rats	(1) Ameliorates PAH through attenuating inflammation and improving endothelial cell survival and function.	Li et al., 2016
	(2) Animal; Cell line	(2) HPSMCs; Rats	(2) Regulates hypoxia-mediated human pulmonary artery smooth muscle cell proliferation, apoptosis and migration via TGF-β1/ERK1/2 signaling pathway.	Li et al., 2017
	(3) Animal/ Primary cells	(3) Rats; Adult Cardiomyocytes, Endothelial Cells and Fibroblasts	(3) Mediates cardioprotective effects through inhibition of ROS production induced by pro-hypertrophic agents via AMPK-mediated activation of anti-oxidant enzymes.	Niemann et al., 2020
	(4) Animal	(4) Rats	(4) Mitigate the progression of arteriovenous shunt-induced pulmonary artery hypertension in rats.	Guan et al., 2021

*AdipR1, adiponectin receptor 1; ADSCs, human adipose derived mesenchymal stem cells; AMPK, AMP-activated protein kinase; CREB, cAMP responsive element binding protein; DsbA-L, disulfide-bond A oxidoreductase-like protein; eNOS, endothelial nitric oxide synthase; ERK1/2, mitogen-activated protein kinase; ERS, endoplasmic reticulum stress; HPSMCs, human pulmonary smooth muscle cells; JNK, c-Jun N-terminal kinase; KO, knock out; LKB1, Serine/Threonine Kinase 11; MD2, Lymphocyte Antigen 96; MI/R, myocardial ischemia/reperfusion; MYD88, MYD88 innate immune signal transduction adaptor; NFκB, nuclear factor-κB; NO, nitric oxide; NLRP3, NLR family pyrin domain containing 3; Nrf2, NFE2 like BZIP transcription factor 2; PAH, pulmonary artery hypertension; PKA, protein kinase cAMP-activated catalytic subunit alpha; PPARγ, peroxisome proliferator activated receptor gamma; ROS, reactive oxygen species; TAC, transverse aortic coarctation; TGF-β1, transforming growth factor beta 1; TLR4, toll-like receptor 4; TNF-α, tumor necrosis factor alpha; VSMCs, vascular smooth muscle cells; WT, wild type.*

CTRP9 plays a pivotal role in the pathogenesis of atrial fibrillation after MI. It was showed that the systemic administration of CTRP9 attenuated atrial inflammation, fibrosis, and vulnerability to atrial fibrillation in post-MI rats (Liu et al., 2019a). Interestingly, Liu et al. (2019a; 2019b) also performed a similar experiment to demonstrate that administration of CTRP9 improves post-MI early cardiac function by regulating M1/M2 macrophage polarization, in agreement with the previous publication. Overexpression of CTRP9 activated PKA to inhibit cardiomyocyte apoptosis, increased the survival rate of mice, restored cardiac function, and reduced myocardial apoptosis and fibrosis (Sun et al., 2013). In a model of transverse aortic coarctation (TAC), CTRP9 was mainly expressed in the endothelial lining of myocardial capillaries. Overexpression of CTRP9 promoted hypertrophic myocardial remodeling and dysfunction after TAC in response to pressure overload, while TAC was alleviated in CTRP9 knockout mice, suggesting that upregulation of CTRP9 promotes maladjusted cardiac remodeling and left ventricular dysfunction (Appari et al., 2017). In contrast, CTRP9-deficient mice show exaggerated cardiac hypertrophy, fibrosis, ERS-initiated apoptosis and oxidative stress compared with HFD-fed wild-type mice, indicating that CTRP9 neutralizes myocardial lipotoxicity and inhibits cardiac hypertrophy (Zuo et al., 2020b).

Although CTRP9 alone cannot promote the cardiogenic differentiation of adipose tissue derived stem cells (ADSCs), combined use with CTRP9 promotes ADSCs proliferation and migration to antagonize hydrogen peroxide-induced cell death (Yan et al., 2017), indicating that CTRP9 protects against oxidative stress-induced cell death. Recently, miR-34a-5p was reported to target and downregulate CTRP9 in cardiomyocytes. Therefore, inhibition of miR-34a-5p may facilitate the protective function of ADSCs against MI damage by stimulating the expression of CTRP9 (Weng et al., 2019). In addition, miR-214-3p was identified as a novel CTRP9 targeting miRNA. The downregulation of miR-214-3p results in elevation of CTRP9 expression, which in turn alleviates cardiac remodeling, including attenuated interstitial fibrosis, improved cardiac function, and enhanced survival rate in rat with MI chronic intermittent hypoxia, suggesting that CTRP9 may be a novel therapeutic target against pathologic remodeling (Du et al., 2020). Furthermore, CTRP9 affects cell survival by regulating oxidative stress-mediated autophagy, thus providing a potential therapeutic target for cardiac lipid toxicity (Zuo et al., 2020a). Together, these data provide additional clarification regarding the regulation of CTRP9.

### Coronary Artery Disease

CTRP9 levels are significantly reduced in female patients with CAD, T2DM and coronary artery disease (CAD) secondary to T2DM compared to healthy subjects. Meanwhile, the expression of CTRP9 was negatively correlated with monocyte chemoattractant protein 1 secretion in patients’ serum (Ahmed et al., 2018). In contrast, levels of circulating CTRP9 were significantly increased patients with T2DM and CAD, suggesting a compensatory response to insulin resistance, inflammatory milieu and endothelial dysfunction (Moradi et al., 2018). These variations may be due to sampling variations and age differences in the cohort in the published reports. Polymorphisms of *CTRP9* gene were associated with increased susceptibility and pathogenesis of CAD. Two single nucleotide polymorphisms (SNPs) were successfully genotyped in CAD patients. The frequency of the AA genotype in CAD patients was lower than that in healthy controls, while CC genotype was higher than healthy controls. These results suggests that the frequency of CC genotype of CTRP9 is correlated with an increased risk of CAD; however, the specific mechanism needs to be studied further (Huang et al., 2020). Auto-antibodies against the second extracellular loop of the β1 adrenoceptor suppressed the expression of CTRP9 in the heart and exaggerated ventricular remodeling (Du et al., 2019). Obstructive sleep apnea is closely related to the incidence and progression of CAD, and CTRP9 levels were negatively correlated with apnea levels; however, it was positively correlated with left ventricular ejection fraction in all participants, indicating that CTRP9 may play a role in the pathogenesis of CAD exacerbated by obstructive sleep apnea (Li et al., 2020). Dramatically, elevated levels of CTRP9 may represent a compensatory response to CAD in these patients. Further studies will be needed to confirm this possibility.

### Atherosclerosis

Clinical studies have shown that plasma CTRP9 levels are associated with an increased risk of atherosclerosis in diabetic patients without chronic kidney disease, but not with obesity, adiponectin, and traditional cardiovascular risk factors, indicating that CTRP9 may play a role in the progression of atherosclerosis in patients with T2DM (Asada et al., 2016). Wang et al. (2015) also found that circulating or expression levels of CTRP9 in epicardial adipose tissue of patients with coronary heart disease were significantly lower than those of healthy controls, confirming that circulating and coronary CTRP9 levels play an important role in inflammation and coronary atherosclerosis in patients with coronary heart disease. In contrast, arterial stiffness is a vascular parameter that predicts cardiovascular events. For instance, brachial-ankle pulse wave velocity is a unique measurement of systemic arterial stiffness, which is measured by waveform analysis of brachial and tibial arteries (Kim et al., 2019). Serum CTRP9 concentration is positively correlated with brachial-ankle pulse wave velocity in patients with T2DM (Jung et al., 2014). Moreover, cardiovascular disease caused by atherosclerosis are the main cause of death in renal transplant recipients (Zanazzi et al., 2010). Changes in serum CTRP9 levels are negatively correlated with the aortic calcification area index. Meanwhile, colocalization of CTRP9 and AdipoR1 was reported in the luminal side of intra-renal arterial intima, and CTRP9 was found to promote the progression of aortic calcification through AdipoR1 (Miyatake et al., 2020), suggesting that CTRP9 prevent the progression of aortic calcification through AdipoR1.

**FIGURE 3:**
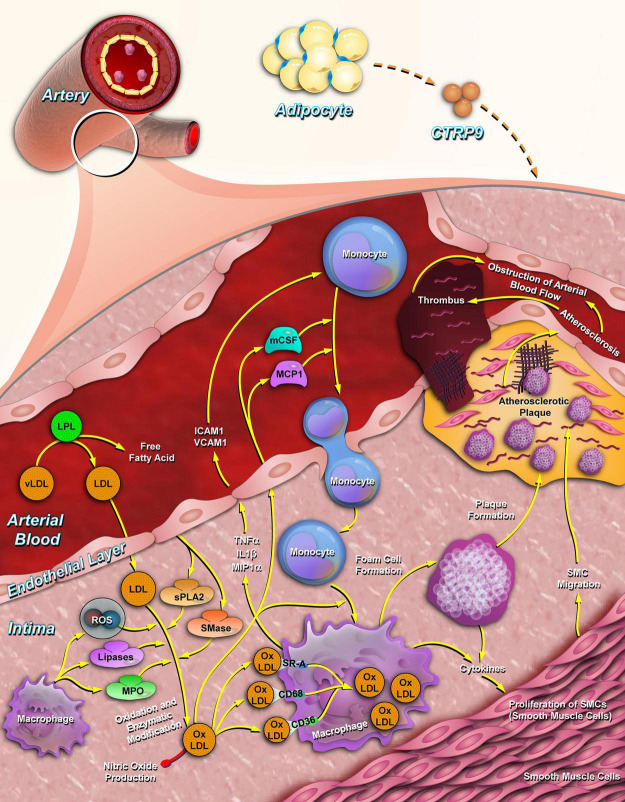
CTRP9 inhibits atherosclerotic plaque formation. CTRP9 is synthesized by adipose tissue (relatively higher expressed in stromal vascular fraction) and secreted into the plasma. In arterial vessels, CTRP9 inhibits the cholesterol oxidation of low-density lipoproteins by lowering the secretion of inflammatory factors, such as IL-6, MCP-1, and TNF-α. CTRP9 reduces the adhesion and migration of monocytes to endothelial cells and promotes the foaming of macrophages by promoting cholesterol efflux from macrophages. CTRP9, C1q/tumor necrosis factor-related protein 9; IL-6, interleukin-6; TNF-α, tumor necrosis factor alpha; MCP-1, monocyte chemoattractant protein-1.

As a unique and pleiotropic adipokine, CTRP9 protects against the development of atherosclerosis through multiple mechanisms (Figure 3; Yu et al., 2018). Animal experiments demonstrated that overexpression of CTRP9 enhances the stability of plaques by reducing the secretion of proinflammatory cytokines from macrophages and inhibited the formation of atherosclerotic plaques after carotid artery constriction in ApoE knockout mice (Li et al., 2015). In addition, CTRP9 overexpression substantially attenuated atherosclerotic lesion size in ApoE knockout mice fed with HFD and reduced the proportion of macrophages in atherosclerotic regions, indicating that CTRP9 exerts a protective role in early atherosclerotic lesions (Huang et al., 2019). There are series *in vitro* experiments to reveal the potential molecular mechanisms. First, CTRP9 exhibited athero-protective function to abolish stimulations by oxidized low-density lipoprotein (ox-LDL) in macrophages (Zhang et al., 2019). In detail, Chen et al. (2020a) determined the anti-inflammatory effect of CTRP9 on Raw 264.7 macrophages and demonstrated that CTRP9 induced an increase in iNOS expression in a time- and dose-dependent manner via activation of JAK2/STAT3 signaling. Second, CTRP9 reduced the number of lipid droplets, lowered the levels of cholesteryl ester, promoted cholesterol efflux in ox-LDL-induced THP1 macrophages, suggesting that CTRP9 protects against atherosclerosis by promoting cholesterol efflux to reduce the formation of foam cells (Zhang L. et al., 2018). Third, overexpression of CTRP9 inhibited the production of reactive oxygen species (ROS) and enhanced mitochondrial biogenesis in human aortic vascular endothelial cells (HAECs) (Scheja and Heeren, 2019). Alternatively, HAECs were pretreated with TNF-α to induce inflammation, then treatment with CTRP9 significantly prevented the activation of NFκB and subsequently increased the phosphorylation of AMPK to reduce the inflammation cytokines in vascular endothelial cells (Wong et al., 2004). These findings are highly consistent with the molecular mechanism observed in RAW 264.7 macrophages induced by ox-LDL (Seldin et al., 2014), suggesting that CTRP9 activates AMPK or prevention of NFκB signaling pathway is required for the reduction of atherosclerotic plaque formation. In addition, incubation with CTRP9 inhibited ox-LDL-impaired endothelial dysfunction, including migration, proliferation, ROS production, apoptosis, angiogenesis and the generation of nitric oxide in human umbilical vein endothelial cells (HUVECs) (Schaffler and Buechler, 2012). Pretreatment of HUVECs with CTRP9 inhibited palmitic acid-induced endothelial senescence by increasing the conversion of LC3-I to LC3-II and decreasing p62 levels in a time- and dose-dependent manner through AMPK activation (Lee et al., 2020). In summary, CTRP9 could be valuable approaches to mitigate vascular inflammation and stabilize atherosclerotic plaques. CTRP9 administration may present a potential therapy for the prevention of adverse cardiovascular events.

The conversion of VSMCs to macrophage-like cells after cholesterol loading is a critical step in the pathogenesis of atherosclerosis. CTRP9 significantly reversed the cholesterol induced secretion of pro-inflammatory factors, monocyte adhesion and cholesterol uptake in VSMCs (Liu et al., 2017). Furthermore, in a macrophages and VSMCs co-culture system, CTRP9 promoted the conversion of macrophages toward the M1 phenotype and downregulated the apoptosis and proliferation of VSMCs induced by the activation of JNK signaling pathway (Chen et al., 2020b), suggesting that CTRP9 may play an important role in inhibiting the transformation of VSMCs into foam cells and involved in the formation of polarized macrophages.

### Pulmonary Arterial Hypertension

Results of *in vitro* and *in vivo* experiments have confirmed that CTRP9 improves pulmonary arterial pressure by reducing inflammation and improving endothelial cells survival and function (Li et al., 2016). Alternatively, CTRP9 strikingly promoted hypoxia-mediated cell apoptosis and prevented cell migration in pulmonary arterial SMCs (Li et al., 2017). To explore the cardio-protective effect on right ventricle hypertrophy and failure, pulmonary artery banding was performed in rats to induce compensatory right ventricle hypertrophy. In agreement with the left ventricle, CTRP9 mediated cardio-protective effects by inhibiting ROS production induced by pro-hypertrophic agents via the AMPK-mediated activation of antioxidant enzymes (Niemann et al., 2020). Moreover, we have previously revealed that CTRP9 mitigates the progression of arteriovenous shunt-induced pulmonary artery hypertension (PAH) in rats, including suppressed inflammation, apoptosis and extracellular matrix injury by increased the phosphorylation of Akt and p38-MAPK in the lung tissues of shunt-operated animals, suggesting that CTRP9 maintains the pulmonary vascular homeostasis during the pathogenesis of PAH (Guan et al., 2021). Collectively, the results of the previous study provide novel insight into the mechanisms underlying arteriovenous shunt-induced PAH.

## Pathologies Associated With CTRP9 Expression

### Wound Healing

CTRP9 regulates the growth, differentiation and apoptosis of human keratinocytes, enhances the binding activity of the transcription factor activating protein 1 (AP-1) and increases P38 phosphorylation to enhance the expression of transforming growth factor beta 1 in a dose-dependent manner, thus stimulating wound healing of keratinocytes (Jung et al., 2017). Systemic sclerosis (SSc) is a multisystem disease with variable presentations, organ involvement, and rates of progression (Schinke and Riemekasten, 2019). Levels of CTRP9 is elevated in SSc, which is significantly associated with SSc-associated interstitial lung disease (Korman et al., 2018), which indicate that CTRP9 elevated in patients with SSc independent of disease duration and may be part of a protective response in lung injury.

### Apoptosis

miR-31 negatively regulates the expression of CTRP9 and the disorder of the ceramide (CER) channel during host-pathogen interactions, which leads to caspase-3 and caspase-8 dependent apoptosis, providing a novel therapeutic perspective of CTRP9 on the CER channel formation (Shao et al., 2017). Targeted therapy of neuronal apoptosis after intracerebral hemorrhage may be an important treatment strategy for patients with intracerebral hemorrhage (Salihu et al., 2016). In an intracerebral hemorrhage model induced by injecting bacterial collagenase into the striatum, Zhao et al. (2019) reveled that CTRP9 inhibitneuronal apoptosis via the AdipoR1/ phosphoinositide-3-Kinase, catalytic, delta polypeptide/Akt signaling pathway, suggesting that administration of CTRP9 may be a promising therapeutic strategy in intracerebral hemorrhage management.

### Mesenchymal Stem Cells

Mesenchymal stem cells (MSCs) present a promising treatment strategy for ischemic brain injury (Bonsack et al., 2020). However, with age, the therapeutic potentials of MSCs gradually decreases, thus limiting their therapeutic effects on post-stroke brain repair (Bagheri-Mohammadi, 2020). CTRP9 exert a significant anti-senescence effect in aged MSCs by activating peroxisome proliferator activated receptor gamma coactivator 1 alpha/AMPK signaling and decreasing the oxidative response, indicating that the anti-aging potential of CTRP9 can be directed to improve the efficacy of stem cell therapy. Therefore, CTRP9 may play a critical role in preventing cellular senescence by facilitating stem cell rejuvenation (Li et al., 2018).

### Inflammation

CTRP9, an anti-inflammatory factor, is negatively correlated with the production of inflammatory factors in various diseases, such as asthma, neuritis, and diabetic retinopathy. For example, CTRP9 alleviated asthma symptoms in asthma model mice by reducing airway remodeling (Qian et al., 2020). In addition, CTRP9 reduces brain edema and improves brain function by activating AdipoR1/AMPK/NFκB signaling pathway (Zhao L.et al., 2018). Furthermore, CTRP9 inhibited inflammation by reducing the production of proinflammatory cytokines and downregulated the expression of tight junction protein, thereby preventing blood-retinal barrier degradation and vascular leakage in diabetic retinopathy mice (Li et al., 2019). In patients with cerebral infarction treated with cerebrovascular stent implantation, serum CTRP9 levels were negatively correlated with TNF-α and interleukin-6 levels (Pan et al., 2020). In contrast, clinical studies have shown that there is no correlation between serum CTRP9 levels and pro-inflammatory mediators in patients with multiple sclerosis (Rasooli Tehrani et al., 2019); however, the molecular mechanism needs to be further explored.

## Conclusion and Perspective

**FIGURE 4:**
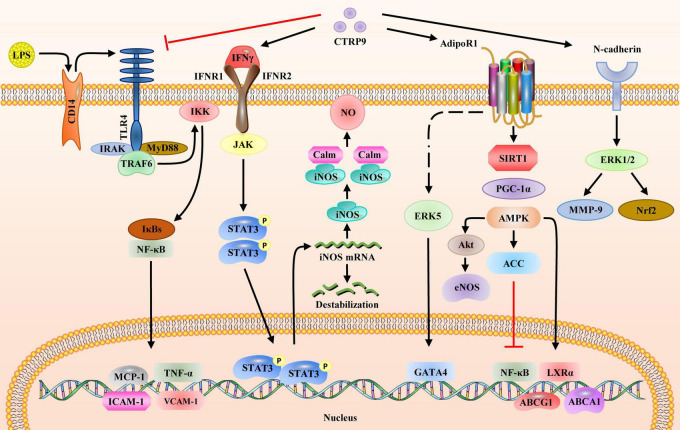
Signaling pathways regulated by CTRP9. CTRP9 binds and affects four receptors on the cell membrane and activates downstream signaling pathways: (1) LPS binds to membrane receptor CD14 to activate the TLR4 signaling pathway. IκBs bind to NFκB to promote the expression of downstream inflammatory genes, including *MCP-1*, *TNF-α*, *ICAM-1*, and *VCAM-1*. CTRP9 blocks the activation of TLR4 receptors via LPS. (2) CTRP9 associates with IFNγ to activate the JAK/STAT3 pathway, thus upregulating the gene and protein expression of iNOS. (3) CTRP9 associates with AdipoR1 to upregulate the SIRT1/PGC1α/AMPK pathway, activate ACC and inhibit the expression of nuclear transcription factor NFκB, upregulate LXRα and promote the expression of ABCA1, and upregulate ABCG1 to activate Akt, thereby increasing eNOS expression; (4) CTRP9 associates with N-cadherin to activate ERK1/2 and upregulate MMP-9 and Nrf2. AMPK, AMP-activated protein kinase; CD14, cluster of differentiation 14; eNOS, endothelial nitric oxide synthase; ERK1/2, mitogen-activated protein kinase; ICAM-1, intercellular adhesion molecule 1; IFNγ, interferon gamma; iNOS, nitric oxide synthase 2; IκB, inhibitor of nuclear factor kappa B kinase subunit beta; JAK, Janus kinase 2; LPS, lipopolysaccharide; LXRα, nuclear receptor subfamily 1 group H member 3; MMP-9, matrix metalloproteinase; NFκB, nuclear factor-kB; Nrf2, nuclear factor, erythroid 2 like 2; PGC1α, peroxisome proliferator activated receptor gamma coactivator 1 alpha; SIRT1, sirtuin 1; STAT3, signal transducer and activator of transcription 3; TLR4, toll-like receptor 4; VCAM-1, vascular cell adhesion molecule 1.

Recent trends in molecular medicine have been focused on dissecting the signaling pathways that control the general functions of different tissues. Understanding these pathways can facilitate the treatment as well as the prevention of multiple pathologies arising due to aberrant molecular mechanisms. CTRP9 has attracted a lot of attention since its discovery in 2009 (Wong et al., 2009). This adipokine is involved in multiple pathophysiological processes associated with various diseases. To summarize, this article reviews recent advances made in demonstrating the link between CTRP9 and a wide set of disorders. The physiological levels of CTRP9 are considerably altered in several diseases (Tables 1–3). Since there is still debate on whether CTRP9 levels should be greater or lower than a specified threshold to effect metabolism, more research is needed to determine whether CTRP9 levels are regulated during the development of obesity and diabetes mellitus. Previous studies reported the key role of CTRP9 in lipid metabolism and cardiovascular disease; however, certain contradictory results need to be discussed and clarified further. First, serum CTRP9 levels are significantly and positively associated with arterial stiffness in patients with T2DM, suggesting that CTRP9 may be a risk factor for angiosclerosis. Second, very little is known about the mechanisms underlying CTRP9 modulation. Identification of key regulators may provide novel insights into the physiological functions of CTRP9 and may facilitate the development of CTRP9-based therapies (Figure 4). Third, CTRP9 expression is correlated with adverse effects. The maximum tolerable dose of exogenous CTRP9 recombinant protein in patients has not yet been thoroughly researched, nor has it been determined whether there are uncontrollable side effects associated with CTRP9 administration. Research on CTRP9, which has considerably enriched our knowledge about the CTRP superfamily, will potentially lead to the development of novel therapeutic strategies. Although more research is needed before CTRP9 can be deemed a viable therapy, our findings should help us get closer to that goal.

## Author Contributions

HG, JF, and QY conceived this manuscript and summarized the contents of the manuscript. HG, YW, and XL collected and prepared the related references. HG and YW drafted the manuscript. HG, XL, and FG drew the figures. All authors contributed to the article and approved the submitted version.

## Conflict of Interest

The authors declare that the research was conducted in the absence of any commercial or financial relationships that could be construed as a potential conflict of interest.

## Publisher’s Note

All claims expressed in this article are solely those of the authors and do not necessarily represent those of their affiliated organizations, or those of the publisher, the editors and the reviewers. Any product that may be evaluated in this article, or claim that may be made by its manufacturer, is not guaranteed or endorsed by the publisher.

## Abbreviations

AdipoR1: adiponectin receptor-1
ADSCs: adipose tissue derived stem cells
Akt: AKT serine/threonine kinase
AMPK: AMP-activated protein kinase
BMI: body mass index
CTRPs: C1q/tumor necrosis factor related proteins
CAD: coronary artery disease
CER: ceramide
ERS: endoplasmic reticulum stress
eNOS: endothelial nitric oxide synthase
HFD: high fat diet
HIIT: high intensity interval training
HOMA-IR: homeostatic model assessment for insulin resistance
HAECs: human aortic vascular endothelial cells
HUVECs: human umbilical vein endothelial cells
IR: insulin resistance
LDL-C: low density lipoprotein cholesterol
MAPK: mitogen-activated protein kinase
MI: myocardial infraction
MI/R: myocardial ischemia/reperfusion
NFκB: nuclear factor-κB
ox-LDL: oxidized low-density lipoprotein
PCOS: polycystic ovary syndrome
ROS: reactive oxygen species
SMCs: smooth muscle cells
SSc: systemic sclerosis
TGF-β1: transforming growth factor beta 1
TAC: transverse aortic coarctation
TNF-α: tumor necrosis factor alpha
VSMCs: vascular smooth muscle cells.
